# Integration of three machine learning algorithms identifies characteristic RNA binding proteins linked with diagnosis, immunity and pyroptosis of IgA nephropathy

**DOI:** 10.3389/fgene.2022.975521

**Published:** 2022-09-28

**Authors:** Xueqin Zhang, Peng Chao, Hong Jiang, Shufen Yang, Gulimire Muhetaer, Jun Zhang, Xue Song, Chen Lu

**Affiliations:** ^1^ Department of Nephrology, People’s Hospital of Xinjiang Uygur Autonomous Region, Urumqi, China; ^2^ Department of Cardiology, People’s Hospital of Xinjiang Uygur Autonomous Region, Urumqi, China; ^3^ Department of Nephrology, The First Affiliated Hospital of Xinjiang Medical University, Urumqi, China

**Keywords:** IgA nephropathy, RNA binding proteins, machine learning, diagnosis, subtypes, immunity, pyroptosis

## Abstract

**Objective:** RNA-binding proteins (RBPs) are essential for most post-transcriptional regulatory events, which exert critical roles in nearly all aspects of cell biology. Here, characteristic RBPs of IgA nephropathy were determined with multiple machine learning algorithms.

**Methods:** Our study included three gene expression datasets of IgA nephropathy (GSE37460, GSE73953, GSE93798). Differential expression of RBPs between IgA nephropathy and normal samples was analyzed *via* limma, and hub RBPs were determined through MCODE. Afterwards, three machine learning algorithms (LASSO, SVM-RFE, random forest) were integrated to determine characteristic RBPs, which were verified in the Nephroseq database. Immune cell infiltrations were estimated through CIBERSORT. Utilizing ConsensusClusterPlus, IgA nephropathy were classified based on hub RBPs. The potential upstream miRNAs were predicted.

**Results:** Among 388 RBPs with differential expression, 43 hub RBPs were determined. After integration of three machine learning algorithms, three characteristic RBPs were finally identified (DDX27, RCL1, and TFB2M). All of them were down-regulated in IgA nephropathy than normal specimens, with the excellent diagnostic efficacy. Additionally, they were significantly linked to immune cell infiltrations, immune checkpoints, and pyroptosis-relevant genes. Based on hub RBPs, IgA nephropathy was stably classified as two subtypes (cluster 1 and 2). Cluster 1 exhibited the relatively high expression of pyroptosis-relevant genes and characteristic RBPs. MiR-501-3p, miR-760, miR-502-3p, miR-1224-5p, and miR-107 were potential upstream miRNAs of hub RBPs.

**Conclusion:** Collectively, our findings determine three characteristic RBPs in IgA nephropathy and two RBPs-based subtypes, and thus provide a certain basis for further research on the diagnosis and pathogenesis of IgA nephropathy.

## Introduction

Immunoglobulin A (IgA) nephropathy is the most frequent form of primary glomerulonephritis globally (Moldoveanu et al., 2021). About one-third of IgA nephropathy patients will develop end-stage renal disease within 20 years after diagnosis by kidney biopsy (Zeng et al., 2021). IgA nephropathy has been an important cause of end-stage renal disease among young adults. The predominant histological characteristics are immune deposits dominated by granular diffuse IgA (primarily comprising polymeric IgA1) in the mesangial region, usually linked to increased mesangial cells along with matrix expansion (Xie et al., 2021). In China, IgA nephropathy occupies 45.26% of primary glomerular diseases, and remains the most common cause of uremia (26.69%) (Li Y. et al., 2021). Currently, the comprehension of the pathophysiology of IgA nephropathy remains undefined, which involves multiple potential players (composed of mucosal immune system, complement system, microbiome, etc.) (Suzuki and Novak, 2021). Nevertheless, the absence of gene models to diagnose IgA nephropathy limits personalized risk-based therapeutic options.

**FIGURE 1:**
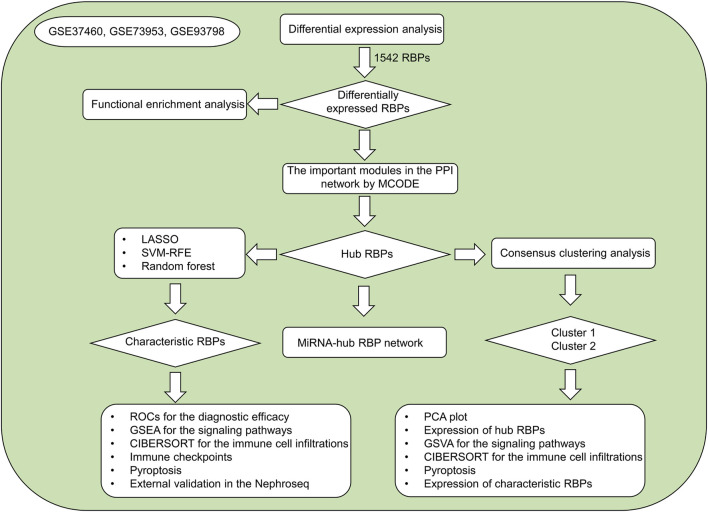
The overall flowchart of our study.

RNA binding proteins (RBPs) exert critical roles in nearly all aspects of cell biology (Sternburg and Karginov, 2020). They orchestrate post-transcriptional regulatory events of gene expression (messenger RNA (mRNA) splicing, RNA stability, translation, etc.) (Wu and Xu, 2022). RBPs act as repressors or activators when interacting with mRNAs, and their binding sites are broad ranging from 5′-UTR to 3′-UTR. The most updated human RBP catalog comprises of 1,542 genes (Supplementary Table S1). Several RBPs have been proven to correlate with innate immune response and various programmed cell death types especially pyroptosis (Zheng and Kanneganti, 2020). Altered expression and dysfunctions of RBPs result in IgA nephropathy progression (Hahn et al., 2010; Wu et al., 2020). In the present study, three machine learning algorithms (LASSO, SVM-RFE, random forest) were integrated to determine characteristic RBPs in IgA nephropathy as well as developed two RBPs-based subtypes, offering a certain basis for further research on the diagnosis and pathogenesis of IgA nephropathy. Figure 1 illustrates the overall design of our study.

## Materials and methods

### Data collection

**FIGURE 2:**
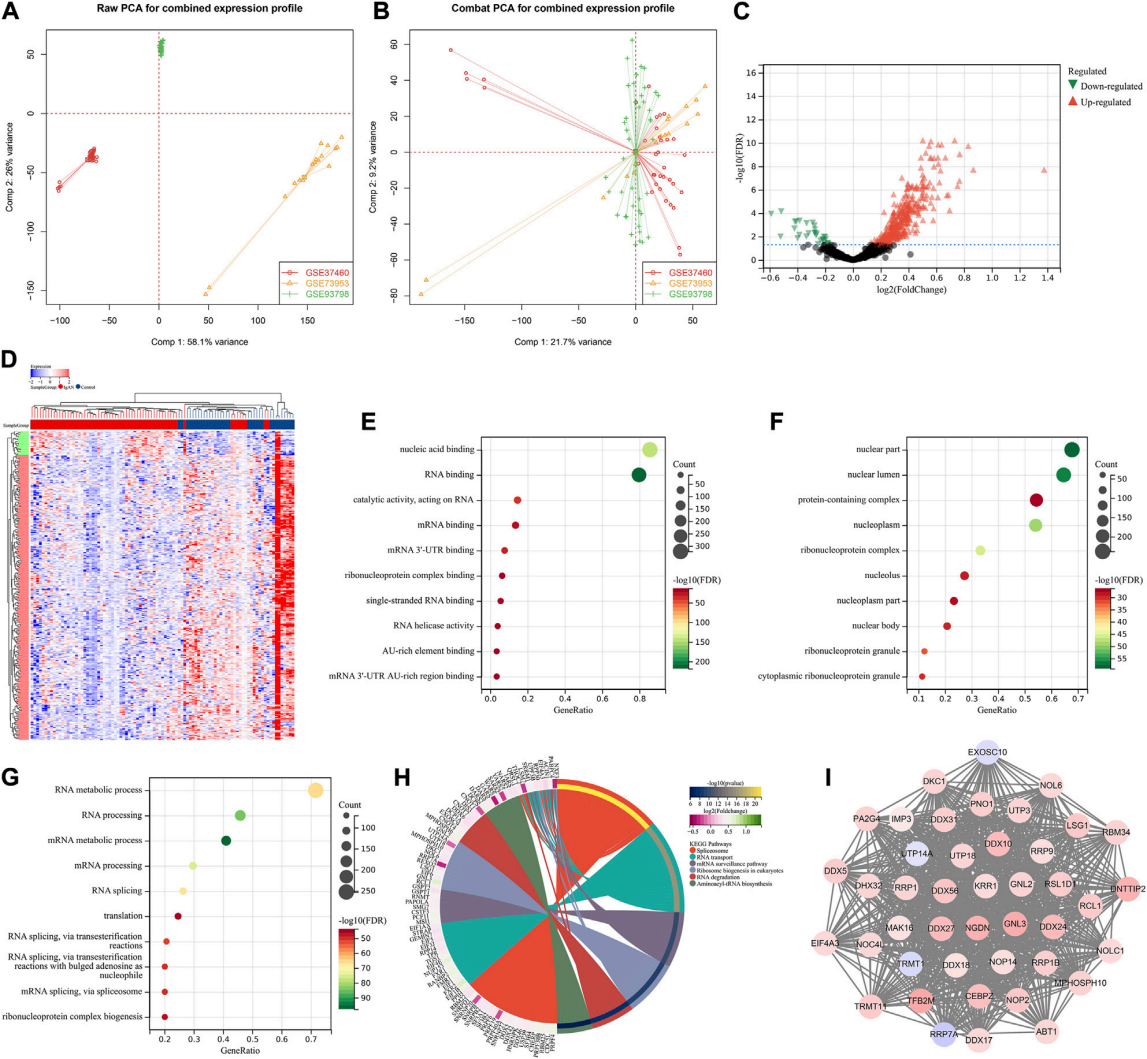
Identification of hub RBPs in IgA nephropathy. **(A, B)** PCA plots for combined transcriptomic profiling of GSE37460, GSE73953, and GSE93798 datasets before and after batch correction. **(C)** Volcano diagram for the up- and down-regulated RBPs in IgA nephropathy than normal samples. Red, up-regulated RBPs; black, not significant RBPs; green, down-regulated RBPs. **(D)** Heatmap of the differential expression of RBPs in IgA nephropathy and normal samples. Red, up-regulation; blue, down-regulation. **(E–G)** The top ten biological processes, cellular components, and molecular functions significantly enriched by RBPs with differential expression. **(H)** KEGG pathways significantly enriched by RBPs with differential expression. **(I)** The important module derived from PPI network of RBPs with differential expression. IgAN, IgA nephropathy.

This study retrospectively included four gene expression datasets of IgA nephropathy from the Gene Expression Omnibus (GEO) repository (https://www.ncbi.nlm.nih.gov/gds/), including GSE37460 (Guo et al., 2019), GSE73953 (Nagasawa et al., 2016), and GSE93798 (Liu et al., 2017). The raw transcriptomic data from the Affymetrix platform were pre-processed with robust multiarray averaging method derived from Affy package (Gautier et al., 2004). The batch effects from different datasets were eliminated by ComBat method from surrogate variable analysis (sva) package (Leek et al., 2012). Figures 2A,B depicted the principal component analysis (PCA) before and after batch correction. Probe IDs were mapped to gene symbols on the basis of the corresponding annotation files, and the expression values of all probes matching the same gene were averaged as the final value. Additionally, we retrieved microRNA (miRNA) expression profiling of IgA nephropathy from GSE25590 dataset (Serino et al., 2012).

### Identification of RBPs with differential expression

Differential expression of RBPs between IgA nephropathy and normal samples was analyzed through linear models for microarray data (limma) (Ritchie et al., 2015). False discovery rate (FDR)<0.05 was set as the cut-off criterion. Gene Ontology (GO) and Kyoto Encyclopedia of Genes and Genomes (KEGG) pathway enrichment analysis of RBPs with differential expression was executed utilizing clusterProfiler package (Yu et al., 2012).

### Analysis of protein-protein interaction (PPI)

Interaction between RBPs with differential expression was probed utilizing the Search Tool for the Retrieval of Interacting Genes (STRING) online database according to the default criteria. Through Cytoscape plugin Molecular Complex Detection (MCODE), the important modules in the PPI network were visualized, and the hub RBPs were identified for subsequent analysis (Shannon et al., 2003).

### Screening characteristic RBPs

Three machine learning algorithms were applied for selecting characteristic RBPs. Hub RBPs were utilized for establishing a penalized multivariate Cox proportional hazard survival model through variable selection on the basis of L1-penalized Least Absolute Shrinkage and Selection Operator (LASSO) regression approach from glmnet package, with 10-fold cross-validation (Goeman, 2010). Support Vector Machine-Recursive Feature Elimination (SVM-RFE) method was employed to search for lambda with the smallest classification error to determine the variables. Random forest algorithm was implemented for generating decision tree forest with 10-fold cross-validation. Characteristic RBPs analyzed by above algorithms were intersected.

### Gene set enrichment analysis (GSEA)

GSEA was executed for determining the significant functional terms between groups (Subramanian et al., 2005). The “c2. cp.kegg.v7.5. symbols.gmt” was downloaded from the Molecular Signatures Database (MSigDB), as the reference gene set (Liberzon et al., 2015). The gene set was regarded as significant enrichment if FDR<0.05.

### Estimation of immune cell infiltrations and immune checkpoints

CIBERSORT, a deconvolution algorithm, was execute to quantify 24 immune cell types *via* applying 547 gene expression signatures (Newman et al., 2015). The permutations were set as 100. Samples with *p* < 0.05 reflected that the deconvolution results were relatively reliable, which were included for subsequent analysis. We also collected common immune checkpoints from published literature (IDO1, LAG3, CTLA4, TNFRSF9, ICOS, CD80, PDCD1LG2, CD70, TNFSF9, KIR3DL1, CD86, PDCD1, LAIR1, TNFRSF8, TNFSF15, TNFRSF14, CD40, TNFRSF4, TNFSF14, HHLA2, CD244, CD27, LGALS9, CD28, CD48, TNFRSF25, CD40LG, VTCN1, CD160, CD44, TNFSF18, BTNL2, TNFSF4, CD200, and NRP1.

### Consensus clustering analysis

Through ConsensusClusterPlus package (Wilkerson and Hayes, 2010), a consistency matrix of IgA nephropathy samples was established based on the expression profiling of hub RBPs according to item subsampling = 0.8, feature subsampling = 0.8, distance = 1—Pearson correlation, iteration = 1,000, and the maximum k value = 9. The optimal number of clusters was determined through consensus CDF, tracking plot, and consensus matrix. Principal component analysis (PCA) was conducted to verify this classification.

### Gene set variation analysis (GSVA)

A single sample gene set enrichment analysis (ssGSEA) was executed for estimating the enrichment score of the specified gene signature utilizing GSVA package, with the “c2. cp.kegg.v7.5. symbols.gmt” as the reference gene set (Hänzelmann et al., 2013). The enrichment score was compared between subtypes *via* limma approach.

### External validation

The expression of characteristic RBPs was externally verified in the Nephroseq database (http://v5.nephroseq.org/) that combines a large number of publicly available renal transcriptome profiling. Associations between characteristic RBPs and clinical features were also evaluated.

### Establishment of a miRNA-hub RBP network

The Encyclopedia of RNA interactomes (ENCORI) integrates eight distinct databases for predicting miRNA-mRNA interactions. Here, four databases (Targetscan, PITA, PicTar, and miRanda) were utilized. MiRNAs were regarded as upstream miRNAs of hub RBPs if the results appeared in ≥2 databases. Afterwards, a miRNA-hub RBP network was visualized *via* cytoscape software.

### Statistical analysis

All statistical analysis was executed with R software (version 3.6.3). Continuous variables that fit normal distribution between binary groups were compared utilizing student’s t-test. Otherwise, Mann-Whitney U test was carried out. Receiver operating characteristic curves (ROCs) were plotted to evaluate the diagnostic efficacy of characteristic RBPs in IgA nephropathy. Associations between variables were evaluated with Pearson or Spearman coefficients. The significance was set as *p* < 0.05, and all statistical tests were two-sided.

## Results

### Identification of hub RBPs in IgA nephropathy

A total of 388 RBPs with differential expression were identified according to FDR<0.05 (Figures 2C,D; Supplementary Table S2). GO enrichment results (nucleic acid binding, RNA binding, mRNA binding, AU-rich element binding, mRNA 3′-UTR binding, etc.) revealed the primary biological functions of these RBPs (Figures 2E–G). Additionally, spliceosome, RNA transport, mRNA surveillance pathway, ribosome biogenesis in eukaryotes, RNA degradation and aminoacyl-tRNA biosynthesis pathways were significantly enriched by these RBPs with differential expression (Figure 2H). A total of 43 hub RBPs were identified, including DDX56, UTP3, MPHOSPH10, DDX10, CEBPZ, TRMT11, NOP14, RRP9, PA2G4, NGDN, KRR1, UTP14A, MAK16, UTP18, DDX27, ABT1, DDX24, DNTTIP2, NOP2, IMP3, EXOSC10, DHX32, RRP1, RRP1B, TFB2M, GNL2, RRP7A, NOC4L, LSG1, DDX17, EIF4A3, GNL3, DKC1, RSL1D1, NOL6, DDX31, DDX5, TRMT1, NOLC1, RBM34, RCL1, PNO1, DDX18 (Figure 2I).

### Identification of characteristic RBPs in IgA nephropathy *via* integrating three machine learning algorithms

**FIGURE 3:**
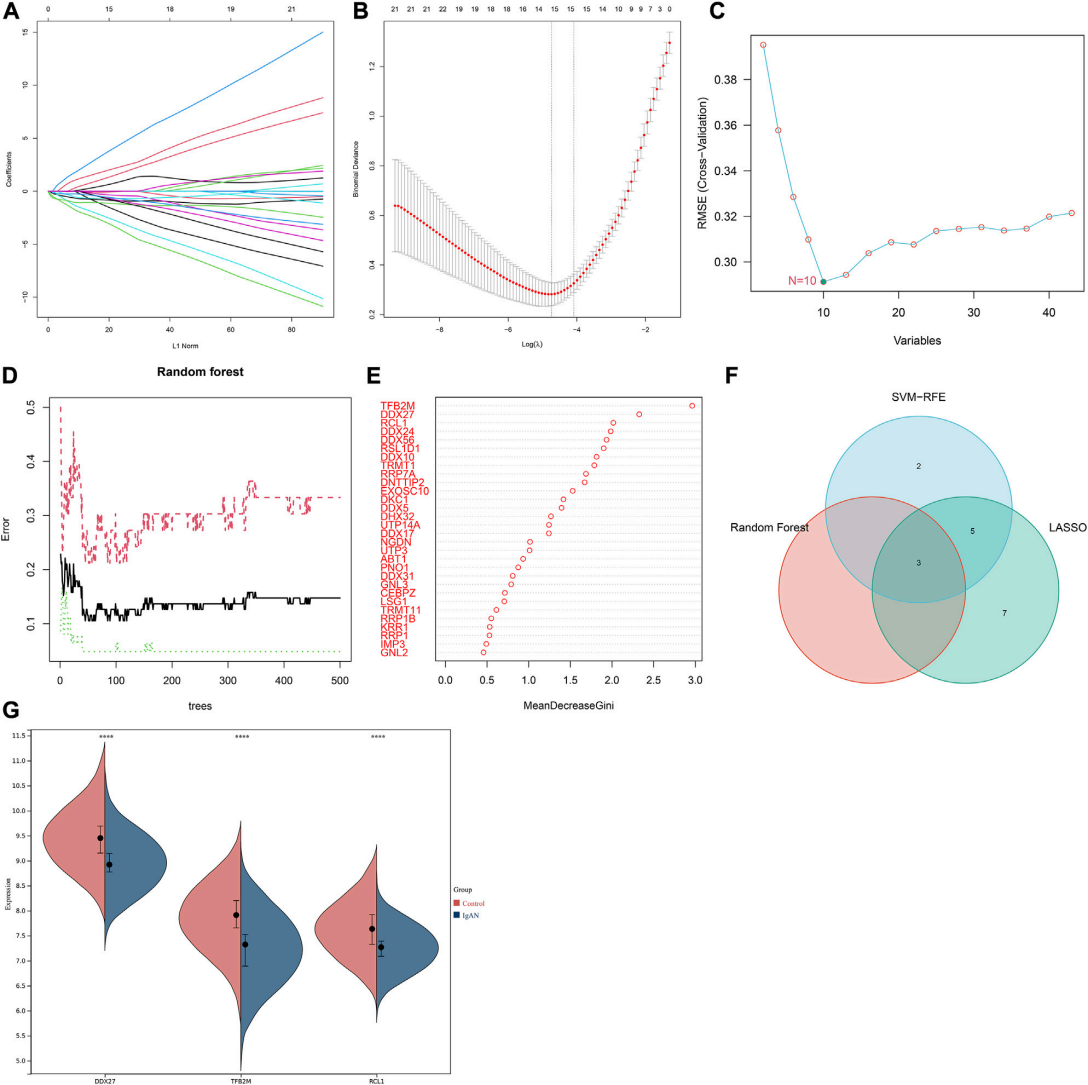
Integration of three machine learning algorithms to determine characteristic RBPs in IgA nephropathy. **(A)** The regression coefficient of each independent variable in the LASSO model. **(B)** Binomial deviance under different log(lambda) in the model. **(C)** SVM-RFE for feature section. **(D)** Random forest for screening characteristic genes. **(E)** The rank of genes according to the relative importance. **(F)** Venn diagram of the intersection results of characteristic genes from three machine learning approaches. **(G)** Expression of characteristic RBPs in IgA nephropathy and normal specimens (****p < 0.0001). IgAN, IgA nephropathy.

Three machine learning algorithms were applied for identifying characteristic RBPs in IgA nephropathy. According to LASSO model, 15 characteristic genes were identified, composed of DDX56, DDX10, UTP14A, DDX27, DDX24, DNTTIP2, IMP3, EXOSC10, TFB2M, RRP7A, GNL3, RSL1D1, DDX31, TRMT1, and RCL1 (Figures 3A,B). SVM-RFE analysis identified 10 characteristic genes including TFB2M, RCL1, RSL1D1, RRP7A, DDX56, DDX27, NGDN, DDX24, TRMT1, and CEBPZ (Figure 3C). Additionally, three characteristic genes were determined based on random forest approach, comprising TFB2M, DDX27, and RCL1 (Figures 3D,E). After integration of above machine learning algorithms, we finally determined TFB2M, DDX27, and RCL1 as characteristic RBPs of IgA nephropathy (Figure 3F). Compared with normal specimens, TFB2M, DDX27, and RCL1 expressions were significantly down-regulated in IgA nephropathy (Figure 3G).

### Characteristic RBPs as reliable diagnostic markers of IgA nephropathy

**FIGURE 4:**
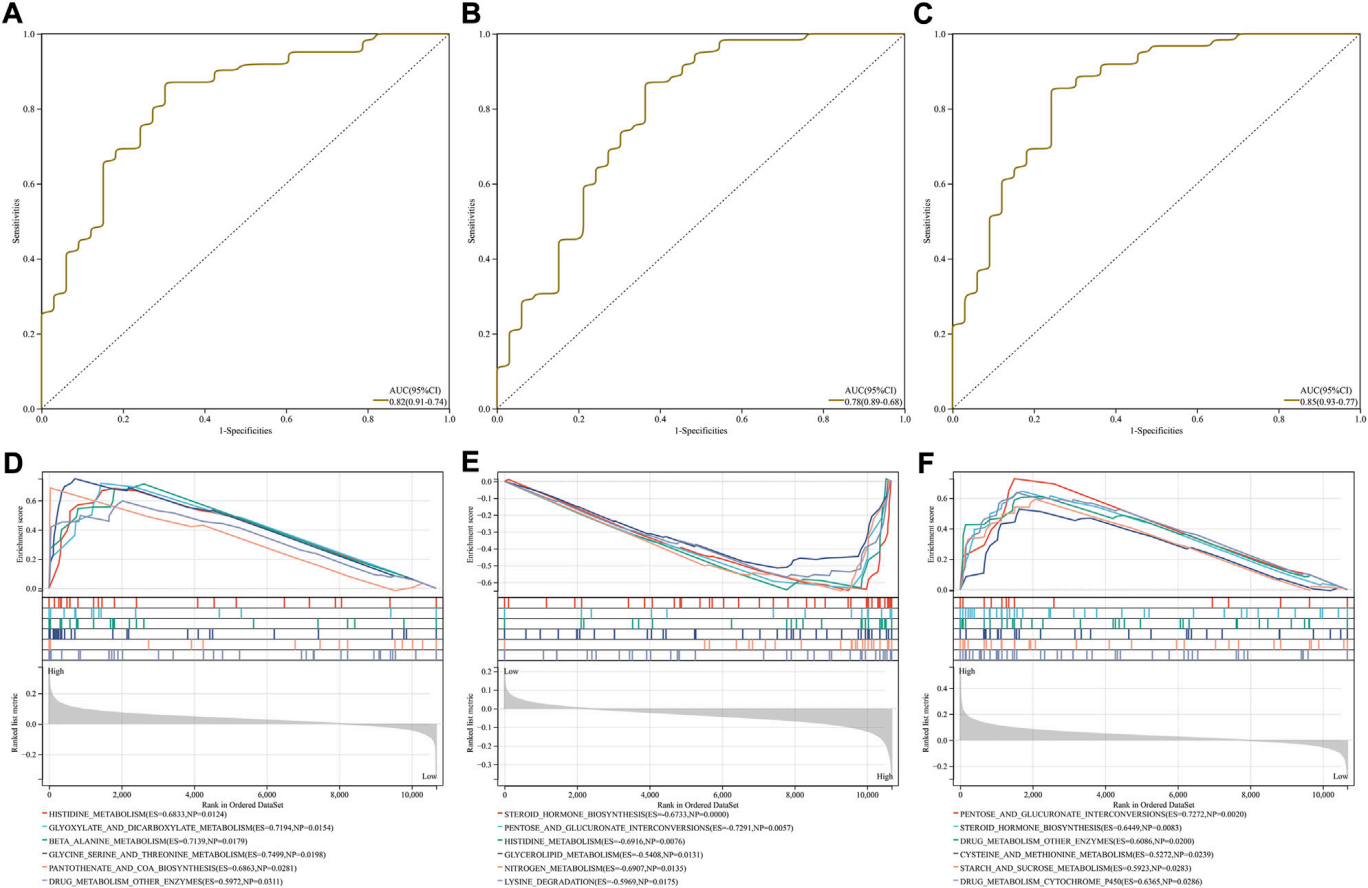
Diagnostic efficacy and involved signaling pathways of characteristic RBPs in IgA nephropathy. **(A–C)** ROCs of **(A)** DDX27, **(B)** RCL1, and **(C)** TFB2M in diagnosing IgA nephropathy. **(D–F)** KEGG pathways involved in **(D)** DDX27, **(E)** RCL1, and **(F)** TFB2M.

To evaluate the diagnostic efficacy of the characteristic RBPs in IgA nephropathy, ROCs were plotted. As a result, the AUC values of DDX27 (Figure 4A), RCL1 (Figure 4B), and TFB2M (Figure 4C) were separately 0.82, 0.78, and 0.85. Above analysis demonstrated that the characteristic RBPs might be reliable diagnostic markers of IgA nephropathy.

### Signaling pathways involved in characteristic RBPs

DDX27 was positively correlated to histidine metabolism, glyoxylate and dicarboxylate metabolism, β-alanine metabolism, glycine serine and threonine metabolism, pantothenate and coA biosynthesis, and drug metabolism other enzymes (Figure 4D). In Figure 4E, RCL1 was negatively linked to steroid hormone biosynthesis, pentose and glucuronate interconversions, histidine metabolism, glycerolipid metabolism, nitrogen metabolism and lysine degradation. Moreover, TFB2M exhibited positive associations with pentose and glucuronate interconversions, steroid hormone biosynthesis, drug metabolism other enzymes, cysteine and methionine metabolism, starch and sucrose metabolism and drug metabolism cytochrome P450 (Figure 4F).

### Characteristic RBPs are linked to immune cell infiltrations and pyroptosis in IgA nephropathy

**FIGURE 5:**
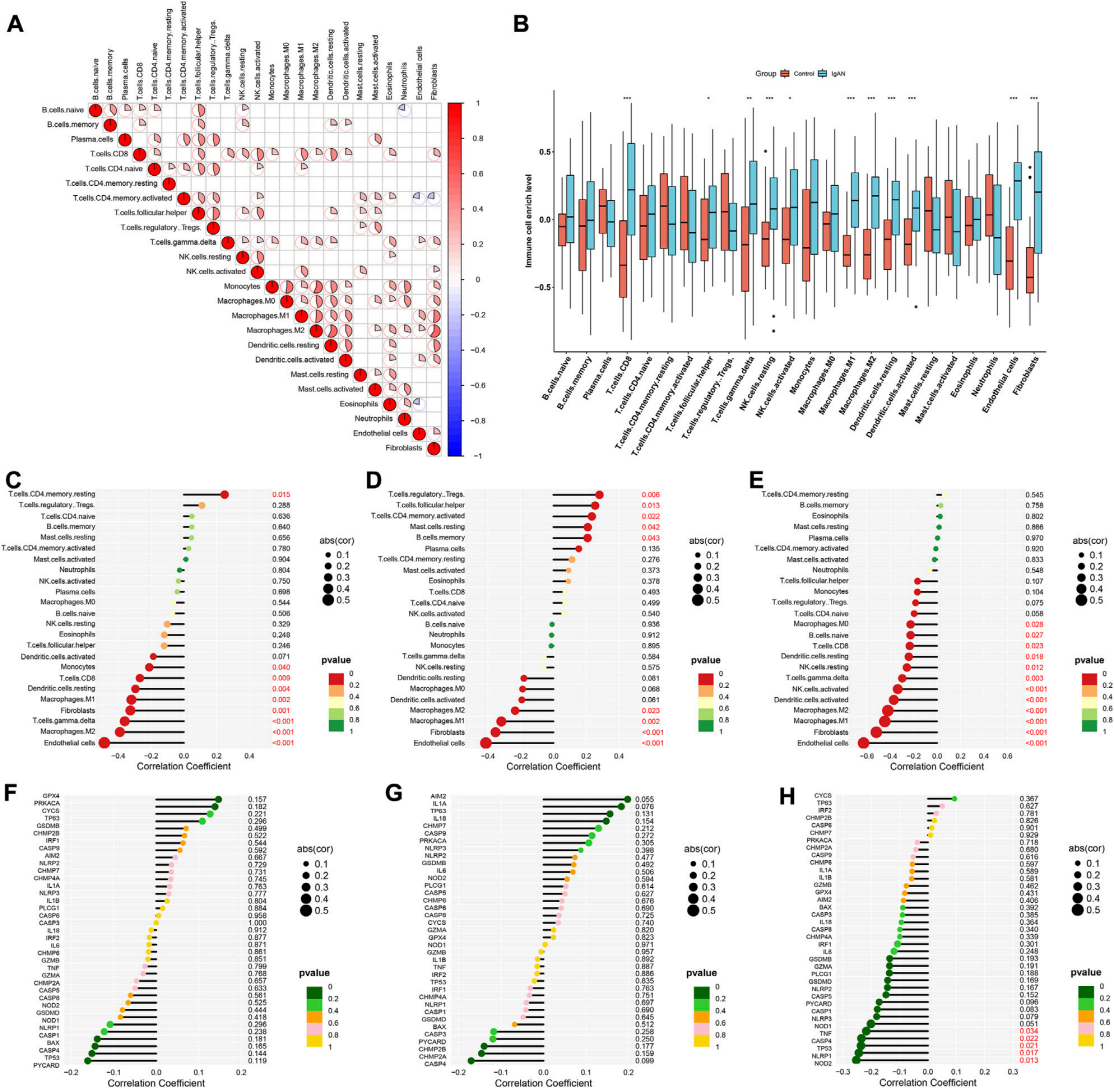
Characteristic RBPs are linked to immune cell infiltrations in IgA nephropathy. **(A)** Correlations between 24 immune cell types in IgA nephropathy specimens. **(B)** Enrichment levels of 24 immune cell types in IgA nephropathy and normal specimens (*p < 0.05; **p < 0.01; ***p < 0.001). **(C–E)** Associations of **(C)** DDX27, **(D)** RCL1, and **(E)** TFB2M with enrichment levels of each immune cell type. **(F–H)** Relationships of **(F)** DDX27, **(G)** RCL1, and **(H)** TFB2M with the expression of pyroptosis-relevant genes. IgAN, IgA nephropathy.

Through CIBERSORT approach, we quantified the enrichment levels of 24 immune cell types. As illustrated in Figure 5A, there were dramatic interactions between immune cells. Compared with normal specimens, CD8 T cells, follicular helper T cells, gamma delta T cells, resting NK cells, activated NK cells, M1 macrophages, M2 macrophages, resting dendritic cells, activated dendritic cells, endothelial cells and fibroblasts exhibited higher enrichment levels in IgA nephropathy (Figure 5B). We also evaluated the correlations between characteristic RBPs and immune cell infiltrations. In Figure 5C, DDX27 was positively associated with memory resting CD4 T cells, but was negatively associated with endothelial cells, M2 macrophages, gamma delta T cells, fibroblasts, M1 macrophages, resting dendritic cells, CD8 T cells, and monocytes. RCL1 exhibited positive interactions with regulatory T cells (Tregs), follicular helper T cells, memory activated CD4 T cells, resting mast cells, memory B cells, but exhibited negative interactions with endothelial cells, fibroblasts, M1 macrophages, and M2 macrophages (Figure 5D). Additionally, TFB2M was negatively linked to endothelial cells, fibroblasts, M1 macrophages, M2 macrophages, activated dendritic cells, activated NK cells, gamma delta T cells, resting NK cells, resting dendritic cells, CD8 T cells, naïve B cells, and M0 macrophages (Figure 5E). Pyroptosis is a type of cell death, that is, crucial for immunity (Niu et al., 2022). Here, the relationships of characteristic RBPs with pyroptosis-relevant genes (DDX27, RCL1, and TFB2M) were analyzed. Among them, TFB2M displayed significantly negative correlations to NOD2, NLRP1, TP53, CASP4, and TNF (Figures 5F–H).

### IgA nephropathy is classified as two subtypes based on hub RBPs

**FIGURE 6:**
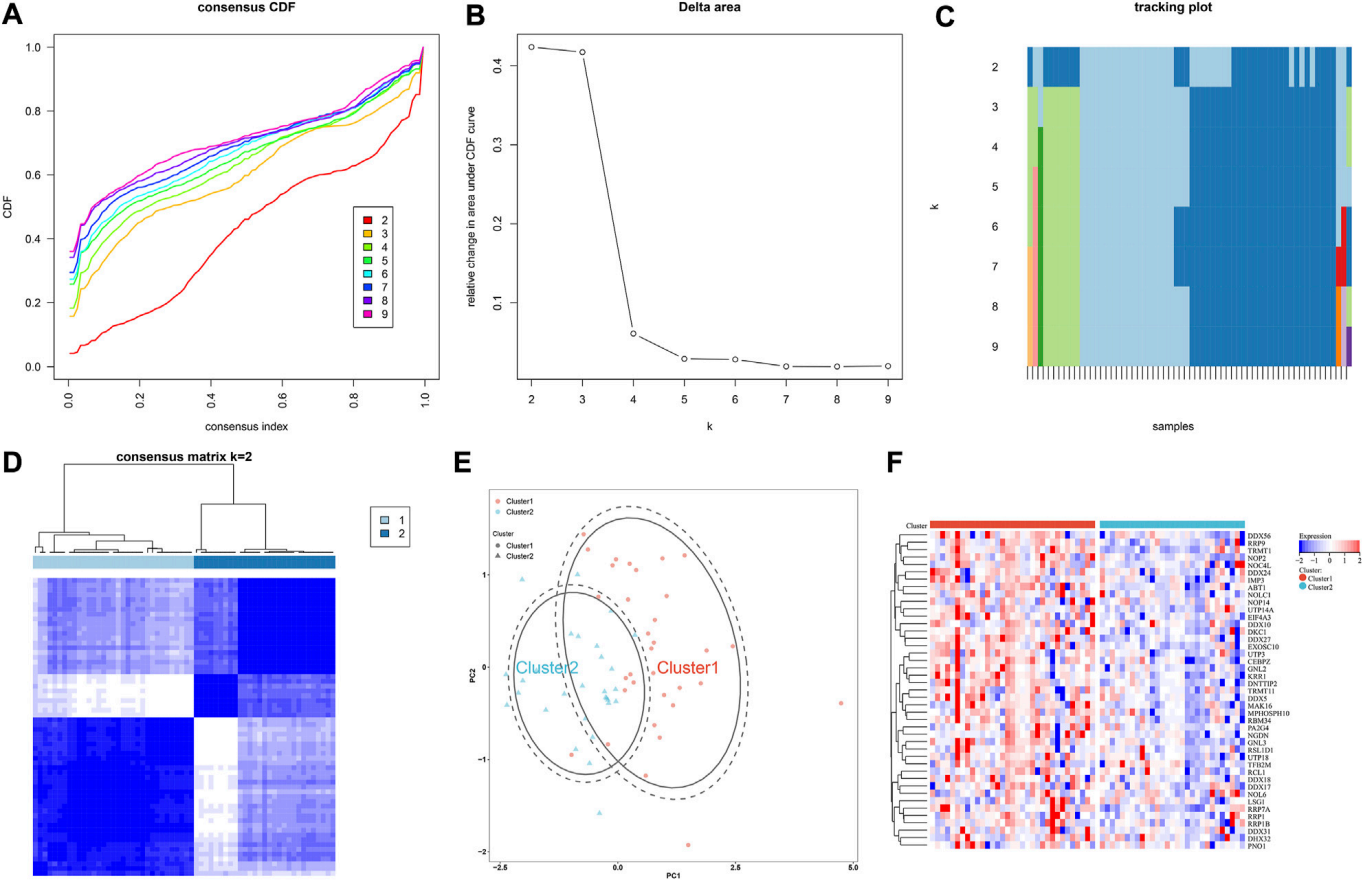
IgA nephropathy is classified as two subtypes based on hub RBPs. **(A)** The consensus CDF across different k values. **(B)** Delta area plot for the relative change in the area under the CDF curves. **(C)** Tracking plot for the item cluster membership across different k values. **(D)** Consensus matrix at k = 2. **(E)** PCA plot of two subtypes. **(F)** Expression of hub RBPs in two subtypes.

Through consensus clustering approach, IgA nephropathy specimens were stably classified as two subtypes, namely cluster 1 and 2 (Figures 6A–D; Supplementary Table S3). PCA plot also demonstrated the difference between subtypes (Figure 6E). Additionally, most hub RBPs exhibited higher expressions in cluster 1 than 2 (Figure 6F).

### Differences in signaling pathways, immune cell infiltrations, immune checkpoints and pyroptosis between hub RBPs-based subtypes

**FIGURE 7:**
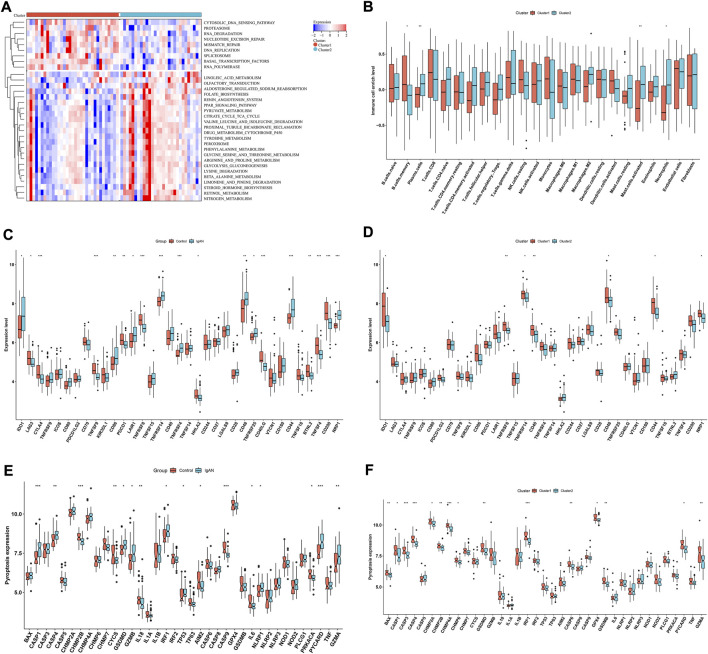
Differences in signaling pathways, immune cell infiltrations and immune checkpoints between hub RBPs-based subtypes. **(A)** Heatmap of the enrichment levels of signaling pathways between subtypes. **(B)** The enrichment levels of 24 immune cell types between subtypes. **(C)** The expression of immune checkpoints in IgA nephropathy and normal specimens. **(D)** The expression of immune checkpoints in two subtypes. **(E)** The expression of pyroptosis-relevant genes in IgA nephropathy and normal specimens. **(F)** The expression of pyroptosis-relevant genes in two subtypes. *p < 0.05; **p < 0.01; ***p < 0.001. IgAN, IgA nephropathy.

In Figure 7A, cytosolic DNA sensing pathway, proteasome, RNA degradation, nucleotide excision repair, mismatch repair, DNA replication, spliceosome, basal transcription factors and RNA polymerase exhibited higher enrichment levels in cluster 1 than 2. Oppositely, linoleic acid metabolism, olfactory transduction, aldosterone regulation sodium reabsorption, folate biosynthesis, renin angiotensin system, PPAR signaling pathway, pyruvate metabolism, citrate cycle TCA cycle, valine leucine and isoleucine degradation, proximal tubule bicarbonate reclamation, drug metabolism cytochrome P450, tyrosine metabolism, peroxisome, phenylalanine metabolism, glycine serine and threonine metabolism, arginine and proline metabolism, glycolysis gluconeogenesis, β-alanine metabolism, limonene and pinene degradation, steroid hormone biosynthesis, retinol metabolism and nitrogen metabolism had higher enrichment levels in cluster 2 in comparison to cluster 1. Compared with cluster 1, cluster 2 was characterized by lower infiltrations of memory B cells, and higher infiltrations of plasma cells, M2 macrophages, activated mast cells and neutrophils (Figure 7B). Additionally, immune checkpoints IDO1, CD86, LAIR1, TNFRSF14, TNFRSF4, CD48, TNFRSF25, CD44, and NRP1 were up-regulated in IgA nephropathy than normal specimens (Figure 7C). Differently, down-regulated LAG3, CTLA4, TNFSF9, PDCD1, TNFRSF8, HHLA2, CD40LG, BTNL2, TNFSF4, and CD200 were found in IgA nephropathy. In Figure 7D, cluster 1 was characterized by higher expressions of IDO1, TNFRSF8, TNFRSF14, CD40, CD48, CD44 and NRP1 than cluster 2. Most pyroptosis-relevant genes exhibited higher expression in IgA nephropathy versus normal specimens (Figure 7E), indicating the activation of pyroptosis pathway in IgA nephropathy. Afterwards, the heterogeneity in pyroptosis was measured between subtypes. In comparison to cluster 1, cluster 2 had lower expression of pyroptosis-relevant genes, indicating higher activity of pyroptosis in cluster 1 (Figure 7F).

### Association of characteristic RBPs with clinical features, hub RBPs-based subtypes and immune cell infiltrations

**FIGURE 8:**
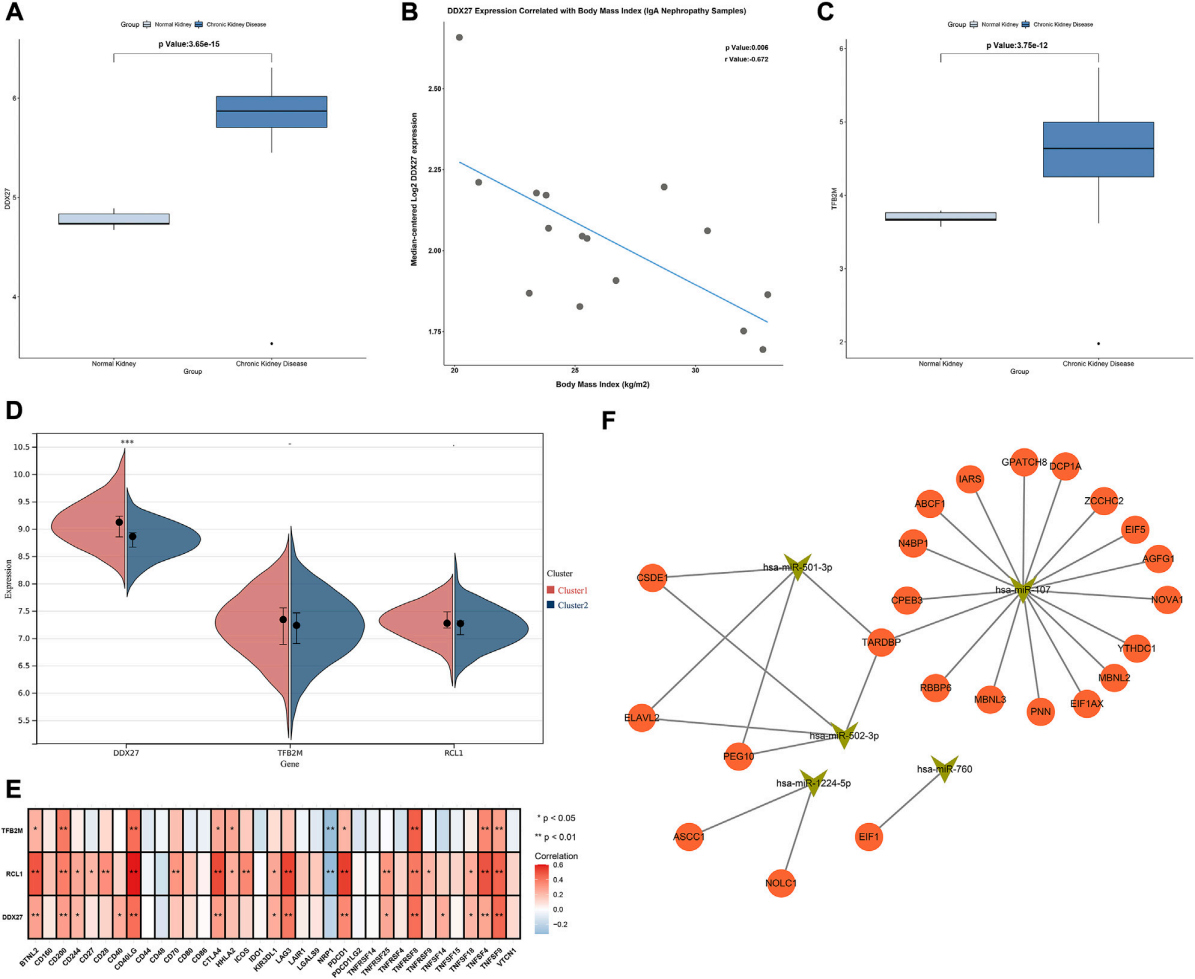
Association of characteristic RBPs with clinical features, hub RBPs-based subtypes, immune cell infiltrations and upstream miRNAs. **(A)** Expression of DDX27 in normal and chronic kidney disease in the Nephroseq database. **(B)** Correlation between DDX27 expression and body mass index of IgA nephropathy samples in the Nephroseq database. **(C)** Expression of TFB2M in normal and chronic kidney disease in the Nephroseq database. **(D)** Expression of characteristic RBPs in two subtypes. **(E)** Heatmap of the associations between characteristic RBPs and immune cell infiltrations in IgA nephropathy. **(F)** The network of characteristic RBPs and upstream miRNAs. *p < 0.05; **p < 0.01.

Characteristic RBPs were further verified in the Nephroseq database. DDX27 was significantly up-regulated in chronic kidney disease than normal kidneys, and was negatively correlated to body mass index (Figures 8A,B). Additionally, up-regulated TFB2M was examined in chronic kidney disease compared with normal kidneys (Figure 8C). Moreover, DDX27, RCL1, and TFB2M expressions were relatively higher in cluster 1 than 2 (Figure 8D). All of them were positively correlated to most immune checkpoints in IgA nephropathy samples (Figure 8E).

### Upstream miRNAs of hub RBPs

We further predicted the upstream miRNAs of hub RBPs. Firstly, we determined miRNAs with differential expression in IgA nephropathy from GSE25590 dataset (Table 1). In Figure 8F, miR-501-3p, miR-760, miR-502-3p, miR-1224-5p, and miR-107 were potential upstream miRNAs of hub RBPs after prediction.

**TABLE 1:** The list of miRNAs with differential expression in IgA nephropathy.

MiRNAs	logFC	Mean expression	t	*p*-value	FDR
miR-107	−2321.43	3942.143	−3.39383	0.005061	0.046251
miR-502-5p	−18.6207	34.11107	−3.49096	0.004216	0.039431
miR-483-5p	−57.0214	62.98214	−3.50524	0.004104	0.038844
miR-362-5p	−92.4143	221.6143	−3.53117	0.003909	0.037443
let-7g	−14493.6	21030.36	−3.57942	0.003571	0.035905
miR-20a	−4168.57	7802.143	−3.57568	0.003596	0.035905
let-7i	−5956.43	9032.5	−3.63129	0.00324	0.033456
miR-374b	−274.786	384.8929	−3.65338	0.003109	0.03333
miR-20b	−621.571	1,068.571	−3.64612	0.003152	0.03333
miR-564	−17.4214	28.675	−3.64029	0.003186	0.03333
miR-765	−23.4164	24.74893	−3.68064	0.002955	0.032779
miR-185	−342.464	552.0536	−3.76167	0.00254	0.029271
miR-134	−38.8429	51.52857	−3.78016	0.002455	0.028697
let-7f	−8376.14	12616.93	−3.87417	0.002062	0.026242
miR-500	−10.2871	18.29929	−3.85099	0.002152	0.026242
miR-132	−33.5157	40.95643	−3.84859	0.002162	0.026242
miR-93	−649.364	998.6036	−3.84438	0.002179	0.026242
miR-15b	−7184.29	12296.43	−3.88947	0.002004	0.026213
miR-500*	−45.7143	64.81429	−3.93033	0.001858	0.025039
miR-22	−2107.14	5074.286	−3.95775	0.001767	0.024474
miR-23a	−2930	5763.571	−3.97519	0.001711	0.024285
miR-103	−4019.43	6833.143	−3.99784	0.001641	0.024155
miR-505*	−24.9971	30.57286	−4.011	0.001602	0.024023
miR-16	−16049.3	31073.93	−4.02855	0.001551	0.023707
miR-155	−412.071	809.75	−4.12935	0.001289	0.020916
miR-98	−165.936	218.1036	−4.23831	0.001057	0.018676
miR-150*	−21.5014	24.05643	−4.27624	0.000987	0.017831
miR-22*	−17.3843	20.66214	−4.31855	0.000914	0.016902
miR-768-5p_v11.0	−247.293	294.6393	−4.47032	0.000696	0.014804
miR-18a	−160.343	218.7	−4.45712	0.000713	0.014804
miR-886-3p	−31.0264	31.83679	−4.44651	0.000726	0.014804
let-7b	−5087.86	6656.071	−4.56311	0.00059	0.013556
miR-221*	−45.1786	62.58929	−4.55652	0.000597	0.013556
miR-365	−42.8286	60.47857	−4.62946	0.000524	0.013304
miR-200b	−14.14	26.88357	−4.61704	0.000536	0.013304
miR-18b	−37.7714	58.52857	−4.68006	0.00048	0.013129
let-7a	−11010.7	15253.21	−4.66164	0.000495	0.013129
miR-24	−3990.71	6935.357	−4.92572	0.000312	0.011569
miR-939	−41.4357	112.7821	−4.84519	0.000359	0.011569
miR-30b*	−11.1014	18.99	−4.82164	0.000374	0.011569
miR-425	−833.143	1,305.571	−4.79374	0.000393	0.011569
miR-760	−11.2556	9.521464	−5.75194	7.84E-05	0.009833
miR-574-3p	−40.0929	48.38929	−5.65536	9.17E-05	0.009833
miR-744	−30.75	36.99643	−5.55679	0.000108	0.009833
miR-671-5p	−25.86	25.20571	−5.3493	0.000152	0.009833
miR-502-3p	−50.0714	69.84286	−5.27775	0.000171	0.009833
miR-1224-5p	−31.5171	31.88429	−5.20559	0.000194	0.009833
let-7d	−2246.86	2828.714	−5.12838	0.000221	0.009833
miR-378*	−40.9929	56.29643	−5.0917	0.000235	0.009833
miR-501-5p	−13.0314	14.50571	−5.09133	0.000235	0.009833
miR-501-3p	−12.5657	13.22	−6.85034	1.45E-05	0.004227
miR-23a*	−11.2007	12.90679	−6.77226	1.62E-05	0.004227
let-7c	−450.336	552.1893	−6.44259	2.66E-05	0.004227

## Discussion

Currently, anatomopathological evaluation of renal biopsies is crucial for diagnosing IgA nephropathy (Li H. et al., 2021). Nevertheless, percutaneous renal biopsies are often not carried out, and proposed histological classifications (Oxford classification system, etc.) have a few shortcomings (Kouri et al., 2021). As the undesirable clinical outcomes of patients with IgA nephropathy is in part the results of delayed diagnosis, reliable non-invasive biomarkers are urgently required, which could be applied to routine clinical practice (Moresco et al., 2015).

Through integration of three machine learning approaches (LASSO, SVM-RFE, random forest), we finally determined three characteristic RBPs of IgA nephropathy: DDX27, RCL1, and TFB2M. All of them displayed remarkable down-regulation in IgA nephropathy. DDX27 is a member of the DEAD-Box nucleic acid helicase family. Previous evidence demonstrates that DDX27 is involved in tumorigenesis. Specifically, DDX27 facilitates hepatocellular carcinoma progression *via* activating ERK signaling (Xiaoqian et al., 2021), and enhances stem cell-like features with undesirable survival outcome of breast cancer (Li S. et al., 2021) and colorectal cancer (Yang et al., 2019). Moreover, it heightens colony-forming capacity of gastric cancer cells and results in terrible survival outcome (Tsukamoto et al., 2015), and strengthens colorectal cancer growth and metastasis (Tang et al., 2018). Other studies demonstrate that DDX27 modulates skeletal muscle growth and regeneration through translational processes (Bennett et al., 2018), and modulates 3’ end generation of ribosomal 47S RNA and stably correlates to the PeBoW-complexing (Kellner et al., 2015). RCL1 is essential for co-transcriptional steps in 18 S rRNA biogenesis (Horn et al., 2011). Evidence suggests that RCL1 weakens hepatocellular carcinoma progression (Jiaze et al., 2022). TFB2M is a mitochondrial transcription factor (Basu et al., 2020), and its C-terminal tail is a part of the autoinhibitory mechanisms that modulate DNA binding (Basu et al., 2020). However, no studies have reported the roles of DDX27, RCL1, and TFB2M in IgA nephropathy. Our ROCs demonstrated the excellent efficacy of these characteristic genes in diagnosing IgA nephropathy.

Our GSEA results demonstrated that DDX27, RCL1, and TFB2M were significantly involved in metabolism pathways such as histidine metabolism, glyoxylate and dicarboxylate metabolism, β-alanine metabolism, glycine serine and threonine metabolism, indicating their crucial roles in RNA metabolism. Most immune cell types exhibited increased infiltration levels in IgA nephropathy, comprising CD8 T cells, follicular helper T cells, gamma delta T cells, resting NK cells, activated NK cells, M1 macrophages, M2 macrophages, resting dendritic cells, activated dendritic cells, endothelial cells, and fibroblasts, consistent with previous research (Chen et al., 2021; Tang et al., 2021). Especially, DDX27, RCL1, and TFB2M were significantly linked to most immune cell populations and immune checkpoints, indicating that above characteristic RBPs might participate in modulating immune cell infiltrations during IgA nephropathy progression. Pyroptosis has gained increasing attention due to its relationship to innate immunity and diseases (Yu et al., 2021). Among characteristic RBPs, TFB2M negatively correlated to several pyroptosis-relevant genes, indicating that TFB2M might modulate pyroptosis pathway during IgA nephropathy.

Genome-wide meta-analysis has uncovered the remarkable molecular heterogeneity across IgA nephropathy patients (Li et al., 2020). Here, based on the hub RBPs, we classified IgA nephropathy two subtypes. Most hub RBPs exhibited higher expressions in cluster 1 than 2. There was the notable heterogeneity in pyroptosis between subtypes, with higher activity of pyroptosis in cluster 1. Additionally, most metabolism pathways displayed higher activity in cluster 2 in comparison to cluster 1. We also noted that cluster 2 had lower infiltrations of memory B cells as well as higher infiltrations of plasma cells, M2 macrophages, activated mast cells and neutrophils. Meanwhile, cluster 1 was characterized by elevated expressions of IDO1, TNFRSF8, TNFRSF14, CD40, CD48, CD44 and NRP1. Altogether, the hub RBP-based subtypes exhibited widespread differences in signaling pathways, immune cell infiltrations and immune checkpoints.

Accumulated evidence shows that miRNAs exert crucial roles in the pathogenesis of IgA nephropathy (Noor et al., 2021; Pawluczyk et al., 2021; Xu et al., 2021). Our further analysis demonstrated that miR-501-3p, miR-760, miR-502-3p, miR-1224-5p, and miR-107 were potential upstream miRNAs of hub RBPs, which might post-transcriptionally regulate the expression of hub RBPs during IgA nephropathy. Despite this, the limitations of our study should be pointed out. First, although we tried our best to collect IgA nephropathy public datasets, the sample size was relatively small. Furthermore, the post-transcriptional mechanisms of hub RBPs will be experimentally verified.

## Conclusion

Collectively, the present study determined three characteristic RBPs as potential diagnostic biomarkers of IgA nephropathy patients through integrating three machine learning approaches (LASSO, SVM-RFE, random forest). Additionally, we classified IgA nephropathy as two RBPs-based subtypes. Altogether, our findings provided a novel clue on the diagnosis and mechanisms of IgA nephropathy.

## Data Availability

The original contributions presented in the study are included in the article/Supplementary Material, further inquiries can be directed to the corresponding authors.

## Abbreviations

IgA, immunoglobulin A; RBPs, RNA binding proteins; mRNA, messenger RNA; GEO, gene expression omnibus; sva, surrogate variable analysis; PCA, principal component analysis; miRNA, microRNA; limma, linear models for microarray data; GO, gene ontology; KEGG, kyoto encyclopedia of genes and genomes; PPI, protein-protein interaction; STRING, search tool for the retrieval of interacting genes; MCODE, molecular complex detection; LASSO, least absolute shrinkage and selection operator; SVM-RFE, support vector machine-recursive feature elimination; GSEA, gene set enrichment analysis; MSigDB, molecular signatures database; GSVA, gene set variation analysis; ssGSEA, single sample gene set enrichment analysis; ROCs, receiver operating characteristic curves.
